# The Web-Based Osteoarthritis Management Resource My Joint Pain Improves Quality of Care: A Quasi-Experimental Study

**DOI:** 10.2196/jmir.4376

**Published:** 2015-07-07

**Authors:** Hema Umapathy, Kim Bennell, Chris Dickson, Fiona Dobson, Marlene Fransen, Graeme Jones, David J Hunter

**Affiliations:** ^1^ Institute of Bone and Joint Research, The Kolling Institute Department of Rheumatology University of Sydney Sydney Australia; ^2^ Centre for Health, Exercise and Sports Medicine Department of Physiotherapy University of Melbourne Melbourne Australia; ^3^ Arthritis Australia Sydney Australia; ^4^ Discipline of Physiotherapy, Clinical and Rehabilitation Sciences Research Group Faculty of Health Sciences University of Sydney Lidcombe Australia; ^5^ Menzies Research Institute Tasmania University of Tasmania Hobart Australia; ^6^ Rheumatology Department Royal North Shore Hospital Sydney Australia

**Keywords:** quality of health care, self-care, osteoarthritis, eHealth, Internet

## Abstract

**Background:**

Despite the availability of evidence-based guidelines for conservative treatment of osteoarthritis (OA), management is often confined to the use of analgesics and waiting for eventual total joint replacement. This suggests a gap in knowledge for persons with OA regarding the many different treatments available to them.

**Objective:**

Our objective was to evaluate outcomes after usage of a Web-based resource called My Joint Pain that contains tailored, evidence-based information and tools aimed to improve self-management of OA on self-management and change in knowledge.

**Methods:**

A quasi-experimental design was used to evaluate the My Joint Pain website intervention over a 12-month period. The intervention provided participants with general and user-specific information, monthly assessments with validated instruments, and progress-tracking tools. A nationwide convenience sample of 195 participants with self-assessed hip and/or knee OA completed both baseline and 12-month questionnaires (users: n=104; nonusers: n=91). The primary outcome measure was the Health Evaluation Impact Questionnaire (heiQ) to evaluate 8 different domains (health-directed activity, positive and active engagement in life, emotional distress, self-monitoring and insight, constructive attitudes and approaches, skill and technique acquisition, social integration and support, health service navigation) and the secondary outcome measure was the 17-item Osteoarthritis Quality Indicator (OAQI) questionnaire to evaluate the change in appropriateness of care received by participants. Independent *t* tests were used to compare changes between groups for the heiQ and chi-square tests to identify changes within and between groups from baseline to 12 months for each OAQI item.

**Results:**

Baseline demographics between groups were similar for gender (152/195, 77.9% female), age (mean 60, SD 9 years) and body mass index (mean 31.1, SD 6.8 kg/m^2^). With the exception of health service navigation, mean effect sizes from all other heiQ domains showed a positive trend for My Joint Pain users compared to the nonusers, although the differences between groups did not reach statistical significance. Within-group changes also showed improvements among the users of the My Joint Pain website for self-management (absolute change score=15%, *P*=.03), lifestyle (absolute change score=16%, *P*=.02), and physical activity (absolute change score=11%, *P*=.04), with no significant improvements for the nonusers. Following 12 months of exposure to the website, there were significant improvements for users compared to nonusers in self-management (absolute change score 15% vs 2%, *P*=.001) and weight reduction (absolute change scores 3% vs –6%, *P*=.03) measured on the OAQI.

**Conclusions:**

The My Joint Pain Web resource does not significantly improve overall heiQ, but does improve other important aspects of quality of care in people with hip and/or knee OA. Further work is required to improve engagement with the website and the quality of information delivered in order to provide a greater impact.

## Introduction

Osteoarthritis (OA) is a highly prevalent and disabling disease and the leading cause of chronic pain [1,2]. It is estimated to affect 1 in 8 adults and the risk for mobility disability due to knee OA is unsurpassed by any other condition in those older than age 65 years [1,3,4]. The aging population and increasing rates of obesity contribute to the estimate that by 2020, the prevalence of OA will have doubled [5].

Numerous evidence-based recommendations for the care of OA that advocate conservative nonpharmacological treatments as the cornerstone of management have been produced to guide health professionals in OA management [6-11]. However, current clinical practice does not reflect these core recommendations and treatment is often limited to analgesics and eventual joint replacement [12-14]. Furthermore, an underuse of key conservative treatments and low levels of referrals to allied health professionals who may provide key lifestyle interventions was reported both internationally and within Australia [12,15].

Surveys of patients with OA reveal a large degree of dissatisfaction with their treatment and a preference for conservative treatment rather than surgery; 81% of patients have indicated that they would not accept surgery if offered and patients with a preference for a treatment most commonly choose physiotherapy [16,17]. The lack of efficacy of current practice coupled with patient dissatisfaction with current care attests to a large and urgent need for people with OA to receive appropriate evidence-based information about treatment options. This need is further supported by patient dissatisfaction with information received from health care professionals, low levels of knowledge regarding OA, and the low levels of patient involvement in treatment decisions [18-20].

The provision of evidence-based information to patients outside the clinical encounter has been long carried through patient education and self-management programs with an aim to result in self-efficacy. Self-management allows patients to recognize problems and play an active role toward improving their condition, essentially being their own caregiver [21].

Consumer-focused strategies are essential for chronic disease self-management to improve outcomes. Although education is a core component of treatment, a 2014 Cochrane review found that current self-management programs for OA offer little benefit and recommended investigation of alternate models of delivery [22]. The potential utility of eHealth for delivery of consumer-focused strategies is increasingly being recognized in-line with the rapid explosion of such technology across all socioeconomic levels and ages [23,24]. It has been suggested in a consensus rating that eHealth interventions for chronic disease management may have greater reach, adoption, implementation, and long-term effectiveness over traditional face-to-face intervention modalities and thereby greater public health impact [25].

This study reports several outcomes following the use of a publicly available Web-based resource, My Joint Pain, over a 12-month period among participants with hip and/or knee OA. My Joint Pain was designed to be a freely available hub of evidence-based information and self-management support resources. The website aims to empower and improve informed treatment decision making in the health care setting. As such, the aim of this study was to evaluate outcomes in users of My Joint Pain on the quality of care and self-management in people with hip and/or knee OA.

## Methods

### Study Design

The evaluation of My Joint Pain [26] was a quasi-experimental study that involved a group of users of the website and a group of nonusers with OA of the hip and/or knee. Outcome measures were administered at baseline and at 12-month follow-up with pre-post changes within each group and a comparison of changes between groups evaluated. The study was approved by the Human Research Ethics Committee of the Universities of Melbourne and Sydney and all participants provided online informed consent.

### Participants

A nationwide convenience sample of adults aged 50 years and older with self-assessed OA in at least one hip or knee joint, access to the Internet, and an email account were recruited using an online strategy involving advertisements placed on various websites, including Arthritis Australia; Melbourne Physiotherapy Department; Centre for Health, Exercise and Sports Medicine; and the Sydney Medical School. Interested participants were directed to a validated screening tool to assess their eligibility. The screening tool contained a series of weighted questions that produced a likelihood of OA score for a nominated hip or knee joint [27]. Participants who received a score above a minimum risk of OA were eligible to participate.

### Procedure

All communication with participants occurred via email and online. Eligible participants completed baseline surveys to collect outcome measures as well as basic demographics and health services utilization. Participants were then informed the My Joint Pain website was accessible for use if they chose to use it. Participants were then contacted 12 months later to complete follow-up surveys. Additionally at follow-up, participants were required to indicate if they were aware of and had used the My Joint Pain website in the previous 12 months. Participants who indicated usage of the website were classified as the intervention group, whereas all other respondents were classified as nonusers.

### Intervention

An expert content committee composed of leading OA researchers, health professionals, and consumers guided Arthritis Australia in the development of My Joint Pain. The website’s functionality was informed by research outlining the key elements required for effective management of chronic disease. Criteria for judging the quality of patient decision aids, prepared by the International Patient Decision Aids Standards (IPDAS) Collaboration also helped to provide a functional framework for the site [28]. Health information was reviewed in-line with Australia’s National Health & Medical Research Council (NHMRC) Guidelines for Consumer Information. The website also complied with the Health on the Net (HON) code standard, an ethical certification for quality health information on the Internet, developed by the HON Foundation, a nongovernmental organization.

The aim of the My Joint Pain website was to provide a credible, tailored information source with a variety of self-assessment tools to improve disease knowledge and self-management of OA. The resource was developed in collaboration with Arthritis Australia (a charitable not-for-profit organization and Australia’s peak arthritis body) and the Bupa Health Foundation (a leading charitable organization dedicated to health in Australia). The website consists of a public access area as well as a member resource. This website is publicly accessible; access is not restricted to the participants of this study only.

The information on the website predominantly focused on hip and knee OA. The public access area of the website included the following pages:

1. Treatment and Management Options
2. Fact Sheets
3. Health Care Providers
4. Watch and Listen

The “Treatment and Management Options” and “Fact Sheets” pages provided detailed and evidence-based information covering a variety of topics including conservative treatments, such as weight loss and exercise, as well as surgical treatments such as joint replacement and arthroscopy. The “Health Care Providers” page provided the location of relevant health services within a local area in Australia and the “Watch and Listen” page provided videos that included patient narratives and information about surgery.

Website visitors could choose to complete a validated hip or knee OA risk assessment followed by a visual analog scale for joint pain [27], a cardiovascular risk assessment (as adapted from the Australian Heart Foundation), gastrointestinal (GI) bleeding risk assessment [29], medication and treatment history, and prior consultation with health professionals (in relation to OA). An OA management algorithm based on the answer provided then created a customized management plan for each user.

After creating an account, the members’ area reinforced the user’s OA management plan and included tailored messages and prompts to encourage users to manage their disease. Weekly, monthly, and biannual assessments allowed members to track pain, weight, treatments and medications, function, and quality of life. The assessments required users to report their pain levels and weight weekly. In addition to these measures, users reported their medication and treatments, completed the Hip disability and Osteoarthritis Outcome Score (HOOS) [30] or the Knee injury and Osteoarthritis Outcome Score (KOOS) [31] and the Quality of Life questionnaires monthly [32]. At 6 months, a Depression, Anxiety and Stress (DASS) [33] assessment and a reevaluation of cardiovascular and gastrointestinal bleeding risk was carried out. This information was processed by an algorithm designed for the website to tailor outputs, such as the management plan, messages that users received based on their needs, and a detailed report that could be discussed with their health care team.

### Outcome Measures

#### Overview

Two reliable and validated tools were used in this study and are described in detail subsequently: the Health Evaluation Impact Questionnaire (heiQ) and the Osteoarthritis Quality Indicator (OAQI).

#### Health Evaluation Impact Questionnaire

The heiQ is designed to evaluate the effects of health education/self-management programs in terms of health education impact and psychosocial impacts [34]. The 40-item questionnaire is categorized into the following 8 independent domains:

1. Health-directed activity,
2. Positive and active engagement in life,
3. Emotional distress,
4. Self-monitoring and insight,
5. Constructive attitudes and approaches,
6. Skill and technique acquisition,
7. Social integration and support, and
8. Health service navigation.

These various domains are defined to evaluate different aspects of health management including change in behaviors, attitudes and viewpoints, and disease management skills. Imperative to this study, the heiQ evaluated applied knowledge in skill and technique acquisition and self-management in health-directed activity, positive and active engagement in life, and self-monitoring and insights [34].

This instrument identifies motivation of change, adherence with management recommendations, coping, and empowerment.

The heiQ is formatted using a Likert scale with options being strongly disagree, disagree, agree, and strongly agree. For analysis purposes, these were numerically labeled as 1, 2, 3, and 4, respectively. The score for each domain was calculated as a mean of the contributing questions with higher scores indicating better outcomes for each domain. All questions in the evaluation were asked in the positive with the exception of emotional distress for which lower scores indicated a more favorable outcome. The difference between the means at follow-up compared to baseline provided the change score.

#### Osteoarthritis Quality Indicator

The OAQI is used to assess the appropriateness of care received by respondents through questions that evaluated patient education and information, clinical assessments, referrals, and treatments [35]. The 17-item tool provides summary pass scores for each item as well as subgroups, which included core treatments versus adjunct treatments and pharmacological treatments versus nonpharmacological treatments (Textbox 1). The OAQI provides insight on adherence to clinical recommendations, aids in identifying barriers in information, and is used in this study to assess change in knowledge acquisition.

Responses to the OAQI were limited to yes, no, and “don’t remember,” and pass rates were represented as a percentage of positive responses out of all eligible responses (yes and no responses). Pass rates were calculated for each variable for the study sample as a whole and summary pass rates were calculated for each participant for core treatments, adjunct treatments, pharmacological treatments, and nonpharmacological treatment.

To examine the 17 variables within each group and between the intervention and nonparticipating group, the change between follow-up and baseline was tabulated for each participant to indicate negative change, no change, or positive change to provide qualitative change indicators.

### Statistical Analysis

#### Overview

Sample size calculations were based on the primary outcome measure, heiQ, with prior data indicating the difference in the response of matched pairs was normally distributed with a standard deviation of 1.2. To reject the null hypothesis that the response difference was zero with a power of 0.9, 95% significance, and true difference in mean response of 0.6, we needed to recruit 44 participants. However, because this investigation was part of a larger study, recruitment was based on other outcome measures and 300 participants were invited to take part in the study.

Before analysis, normality of data was assessed and participants who had not used the website were identified as nonusers.

#### Health Evaluation Impact Questionnaire

Changes within the nonusers and the intervention group were analyzed using a paired *t* test comparing follow-up to baseline scores. Differences in change between the groups were compared using an independent *t* test.

#### Osteoarthritis Quality Indicator

Chi-square tests were used for the evaluation of each individual item within and between the study groups using the qualitative change indicators. The chi-square evaluation for changes within the group compared changes between yes and no responses at baseline and at 12 months. The comparison between groups used the qualitative change indicators (as described previously) in a chi-square evaluation against usage of the website.

Evaluation of the subgroups (core treatments vs adjunct treatments and pharmacological treatments vs nonpharmacological treatments) were carried out using independent *t* tests on each participants change score for each subgroup separately with the use of the website as the independent variable.

**Osteoarthritis Quality Indicator subgroups of domains for core treatments versus adjunct treatments and pharmacological treatments versus nonpharmacological treatments.**

Core vs Adjunct Treatments

Core treatments:

- Disease development
- Treatment alternatives
- Self-management
- Lifestyle
- Physical activity
- Referral physical activity
- Weight reduction
- Referral weight reduction

Adjunct treatments:

- Functional assessment
- Walking aid assessment
- Other aids assessment
- Acetaminophen
- Stronger pain killers
- Nonsteroidal anti-inflammatory drugs (NSAIDs)
- Cortisone
- Referral to orthopedic surgeons

Pharmacological vs Nonpharmacological Treatments

Pharmacological treatments:

- Acetaminophen
- Stronger pain killers
- NSAIDs
- Cortisone

Nonpharmacological treatments:

- Disease development
- Treatment alternatives
- Self-management
- Lifestyle
- Physical activity
- Referral physical activity
- Weight reduction
- Referral weight reduction
- Functional assessment
- Walking aid assessment
- Other aids assessment

## Results

### Participants

A total of 277 eligible participants provided consent and completed baseline measures. As anticipated, a large attrition of volunteers took place and only 195 participants (70.4%) completed the 12-month follow-up questionnaires. Demographic information collected (age, gender, body mass index [BMI], affected joint) showed no significant difference between all collected questionnaires (N=277 including noncompleters), the intervention group (n=104), or the nonusers group (n=91) at baseline (Table 1).

**Table 1:** Demographic characteristics of participants at baseline (N=277).

Characteristics		Baseline N=277	Noncompleters n=82	Nonusers n=91	My Joint Pain users n=104	*P*
Age (years), mean (SD)		61.0 (8.6)	61.7 (8.5)	60.9 (9.1)	60.5 (8.3)	.64
Female, n (%)		212 (76.5)	60 (73)	73 (80)	79 (76.0)	.54
BMI (kg/m^2^), mean (SD)		31.0 (6.8)	30.4 (6.8)	32.5 (10.0)	30.4 (6.8)	.08
**Joint, n (%)** ^a^						.20
	Knee	137 (49.4)	45 (55)	38 (42)	54 (52.9)	
	Hip	43 (15.5)	7 (9)	16 (18)	20 (19.2)	
	Both	92 (33.2)	29 (35)	34 (37)	29 (27.9)	

^a^ Total percentage may not add up as it was possible to receive a risk of OA without indicating a joint.

### Health Evaluation Impact Questionnaire

Within the intervention group, all domains examined for the heiQ except emotional distress, constructive attitudes and approaches, and health service navigation showed significant improvements from baseline to follow-up. Although differences in baseline mean scores between the 2 groups were minimal (difference in means=0.02) and no significant change was observed in the users, a significant deterioration in emotional distress was observed in the nonusers over the 12-month period (mean difference from baseline to follow-up=0.161, *P=*.008) (Table 2).

**Figure 1:**
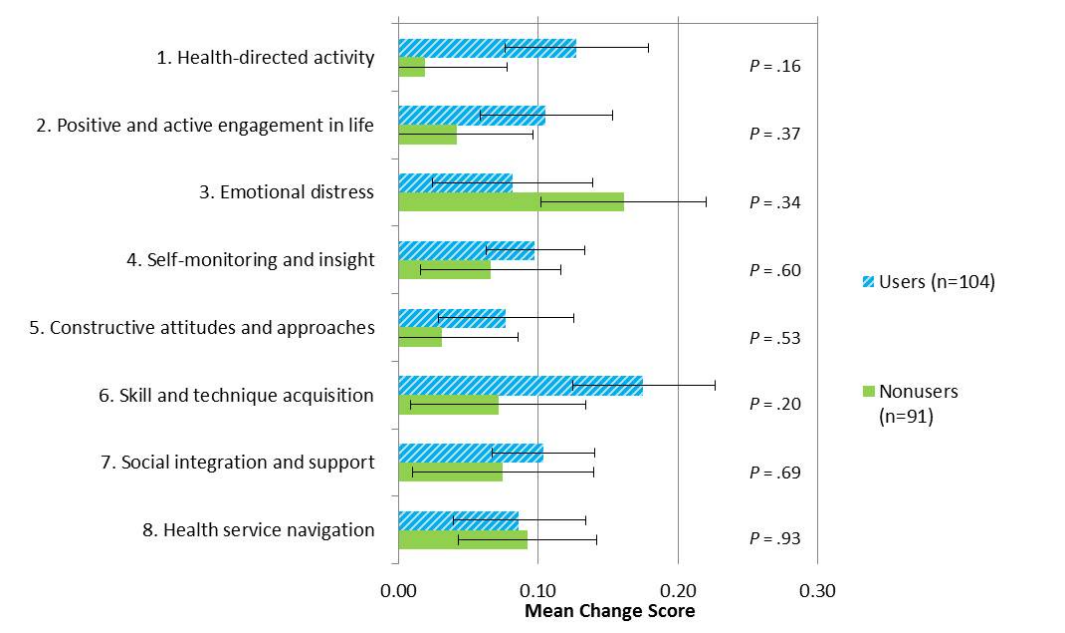
Mean change scores (standard error) between users and nonusers of the My Joint Pain website over a 12-month period for each Health Evaluation Impact Questionnaire domain.

All domains, except health service navigation, showed a clear positive trend in change scores (the difference between follow-up and baseline) in the intervention group relative to nonusers, although the differences in change between the groups were not statistically significant (Figure 1). Mean change scores were calculated as the follow-up score minus the baseline score within each domain and within each group. Positive change scores indicated improvement. *P* values were the result of independent *t* tests comparing the change between groups. All questions in the evaluation were asked in the positive with the exception of emotional distress for which lower scores indicated a more favorable outcome. The improvements appeared particularly pronounced in health-directed activity (*P=*.16), emotional distress (*P=*.34), and skill and technique acquisition (*P=*.20), but none met statistical significance.

**Table 2:** Mean within-group change between 12-month follow-up and baseline for each Health Evaluation Impact Questionnaire domain for users and nonusers of the My Joint Pain website.

heiQ Domains	Users (n=104)			Nonusers ( n=91)		
	Baseline score, mean (SD)	Mean difference (95% CI)	*P*	Baseline score, mean (SD)	Mean difference (95% CI)	*P*
1. Health-directed activity	2.78 (0.71)	0.13 (0.03, 0.23)	.02	2.93 (0.68)	0.02 (–0.10, 0.14)	.74
2. Positive and active engagement in life	2.97 (0.56)	0.11 (0.01, 0.20)	.03	3.10 (0.58)	0.04 (–0.07, 0.15)	.44
3. Emotional distress	2.58 (0.63)	0.08 (–0.03, 0.20)	.16	2.60 (0.82)	0.16 (0.04, 0.28)	.008
4. Self-monitoring and insight	3.05 (0.35)	0.10 (0.03, 0.17)	.01	3.12 (0.43)	0.07 (–0.03, 0.17)	.19
5. Constructive attitudes and approaches	2.99 (0.50)	0.08 (–0.02, 0.17)	.12	3.06 (0.57)	0.03 (–0.08, 0.14)	.58
6. Skill and technique acquisition	2.69 (0.48)	0.18 (0.07, 0.28)	.001	2.79 (0.49)	0.07 (–0.05, 0.20)	.26
7. Social integration and support	2.66 (0.50)	0.10 (0.03, 0.18)	.006	2.64 (0.60)	0.08 (–0.05, 0.20)	.25
8. Health service navigation	2.84 (0.52)	0.09 (–0.01, 0.18)	.07	2.98 (0.59)	0.09 (–0.01, 0.19)	.07

### Osteoarthritis Quality Indicator

Pre-post analysis within the intervention group showed significant improvements in self-management (change score=15.2%, *P=*.03), lifestyle (change score=16.2%, *P=*.02), and physical activity (change score=10.8%, *P=*.04) following 12 months’ exposure to the website. No significant changes were observed within the nonusers group during this period of time. Compared to nonusers, the intervention group showed significant improvements in self-management (change scores 15.2% vs 1.7%, *P=*.001) and weight reduction (change scores 2.5% vs –6.3%, *P=*.03) measured on the OAQI after 12 months (Table 3).

Summary change scores for treatment categories were also obtained. Between the intervention group and nonusers, there were minimal differences that did not reach statistical significance in core treatments (change scores 10.3% vs 4.7%, *P*=.17), adjunct treatments (change scores 3.1% vs 1.2%, *P*=.54), pharmacological treatments (change scores –1.7% vs –2.4%, *P*=.35), and nonpharmacological treatments (change scores 9.5% vs 4.0%, *P*=.29).

**Table 3:** Outcomes of the Osteoarthritis Quality Indicator at baseline and 12 months for users and nonusers of the My Joint Pain website.

OAQI items	Users (n=104)			Nonusers (n=91)			*P*
	Baseline, eligible (pass rate)^a^	12-month follow-up, eligible (pass rate)^a^	*P*	Baseline, eligible (pass rate)^a^	12-month follow-up, eligible (pass rate)^a^	*P*	
1. Disease development	99 (56)	95 (68)	.65	81 (53)	81 (59)	.43	.51
2. Treatment alternatives	99 (62)	100 (65)	.62	88 (52)	86 (56)	.64	.80
3. Self-management	99 (53)	99 (68)	.03	88 (47)	87 (48)	.82	.001
4. Lifestyle	99 (53)	99 (69)	.02	89 (48)	90 (53)	.50	.36
5. Physical activity	103 (77)	104 (88)	.04	90 (79)	91 (86)	.23	.79
6. Referral physical activity	104 (51)	103 (62)	.11	90 (53)	89 (57)	.59	.37
7. Weight reduction	74 (69)	70 (71)	.74	64 (83)	64 (77)	.38	.03
8. Referral weight reduction	68 (18)	66 (24)	.35	64 (25)	57 (39)	.11	.95
9. Functional assessment	89 (47)	76 (50)	.72	76 (45)	76 (51)	.42	.40
10. Walking aid assessment	68 (26)	65 (38)	.14	64 (31)	59 (36)	.61	.22
11. Other aids assessment	55 (15)	55 (18)	.61	58 (17)	51 (27)	.20	.80
12. Pain assessment	103 (64)	100 (67)	.66	91 (62)	88 (53)	.27	.37
13. Acetaminophen	104 (76)	103 (81)	.42	91 (79)	89 (79)	.94	.71
14. Stronger pain killers	94 (62)	92 (67)	.42	80 (59)	75 (52)	.40	.62
15. NSAIDs	83 (78)	83 (76)	.71	64 (77)	55 (80)	.65	.41
16. Cortisone	84 (49)	81 (42)	.38	73 (55)	59 (47)	.40	.06
17. Referral to orthopedic surgeons	73 (53)	81 (58)	.57	66 (52)	59 (53)	.91	.29

^a^ Number of eligible persons calculated as total number of yes and no answers and who did not provide not applicable or “do not remember” answers. Pass rates are the proportion of yes answers over the number of eligible.

## Discussion

### Principal Findings and Comparison

The aim of this study was to evaluate the impact of the My Joint Pain website on self-management and quality of care. Our findings indicated that the resource did not improve all aspects of heiQ or OAQI, but highlighted benefits that included improvements in health-directed activity, positive and active engagement in life, self-monitoring and insights, skill and technique acquisition, and social integration within the heiQ. Improvements in self-management, lifestyle, physical activity, and weight reduction were also observed in the OAQI.

The aging of the population and the growing number of people living with chronic conditions is causing increasing dependence on an overstretched health care system. This burden on the health care system coupled with inadequate attention in the clinical context to OA emphasizes the need for self-management strategies and resources for patients with OA [36]. With current clinical practice in OA being suboptimal and self-management programs being ineffective, identifying alternate self-management resources are important [22,37].

Self-management has been increasingly encouraged and supported in various chronic conditions including diabetes, arthritis, and asthma [38]. Unlike traditional patient education in which patients are provided with disease information, self-management education additionally provides techniques that help patients cope and act with their disease including during periods of more active disease [39]. Increasingly, Web-based self-management information and tools have become available with significant efficacy in various diseases including rheumatoid arthritis, fibromyalgia, and juvenile idiopathic arthritis [40-46]. Evidence-based credible tools for OA are needed and the development of the My Joint Pain website was designed to fill this gap by providing tailored, evidence-based information and tools to improve the self-management of the disease and the quality of the clinical consultation between a patient and members of their health care team.

This study hypothesized that the increased and more tailored information received by the participants in the intervention group would empower them to create a collaborative care plan with their health care team, to input their values in treatment choices, and improve self-management. Our hypothesis was partially supported by the results that showed significant improvements in the quality of care patients received as assessed by the OAQI. Specifically, we saw significant improvements in information received about conservative options, such as self-management, lifestyle, and physical activity, as well as advice regarding weight reduction. These improvements in conservative care knowledge may help to address the existing gap in current clinical practice where management is directed in the first instance to analgesics and then surgery [47]. Furthermore, the improvement in these outcomes could be a result of greater patient confidence in conservative domains that did not require input from a health care professional and where participants could take action independently.

Promising results were also observed when evaluating the impact of the My Joint Pain website with the heiQ. Significant differences in self-monitoring and insights, skill and technique acquisition, and social integration and support were evident within the users of My Joint Pain. Health-directed activity, skill and technique acquisition, and emotional distress were significantly improved for users of the My Joint Pain website and they were the most positively impacted compared to nonusers, although statistical significance was not reached in the differences of change between the groups.

Interestingly, in the heiQ, emotional distress deteriorated in the nonusers but did not change in the intervention group over the 12-month period. This finding contradicted the expectation that improvements would be observed in all domains within the intervention group and none in the nonusers; particularly so in emotional distress as users of the My Joint Pain website had directed encouragement to seek help if biannual check-ups indicated emotional impacts. However, emotional well-being is known to worsen with the progression of OA and it is possible that the website provided a level of maintenance to users to keep their emotional health from deteriorating as observed in the nonusers [48].

The lack of change in the constructive attitude and approaches (indicates how participants view the impact of their disease), and health service navigation domains highlight a gap in the My Joint Pain website. Although there is extensive information on treatments and facts about the disease, there is a lack of information about strategies and techniques to shift ones viewpoint about the impact of OA. In regards to health service navigation, the website does provide a tool to locate nearby health care resources. However, this tool is not easily accessible, well integrated, and not located on the main tool bar, which might account for the observed outcome.

### Addressing Barriers to Self-Management

The gaps highlighted from this study relate to a key barrier of self-management in OA: patient’s perception. Cuperus et al [49] highlighted the role of patient’s perception toward OA, its manageability, the available resources, and their own information needs as both a barrier and a facilitator to self-management. This intervention did not directly aim to modify patient’s perception toward self-management and modification of this gap would require the resource to address the concept of self-management. Alternatively, as suggested by Cuperus et al, encouragement and direction toward the resource from health care professionals would be useful [49].

Other established barriers in self-management include limited resources (financial and transportation), low levels of encouragement and support, and a lack of tailored strategies [50]. The My Joint Pain website addresses these barriers by providing a freely available and easily accessible resource as well as using a variety of instruments to tailor recommendations and encouragement for users. The use of tailored features is also identified as a key pillar for the success of Web-based interventions along with other interactive features including self-management, as addressed in My Joint Pain [51]. The additional pillars that were not addressed include social support and contacts with intervention could be addressed by the inclusion of a forum with other consumers and experts.

Similarly, other studies have addressed the inclusion of these pillars to varying degrees in other Web-based arthritic disease interventions, including self-management education, telerehabilitation, and, most commonly, physical activity. Within Internet-based interventions, self-management was investigated in several studies targeted at rheumatoid arthritis, juvenile idiopathic arthritis, fibromyalgia, and OA [44-46]. These studies showed improvements in quality of life and self-rated global health scores. Studies that included more active elements of an intervention, such as personalized phone calls, forums, and Web conferences, also showed positive improvements [45,46,52,53]. Although these interventions were beneficial, the Internet-based interventions were restricted to participants of the study as opposed to the free, open access provided by the My Joint Pain resource; hence, the results are not directly transferable.

### Limitations

There are several limitations of this study. There was a large attrition of volunteers with almost one-third lost to follow-up. The reduction in sample size prevented further stratified analysis to evaluate outcomes in varying age groups or genders. Furthermore, information was not collected about disease severity and specific usage data, which prevented the study from correlating changes to specific content or frequency of use of the website. This information would have aided more effective redevelopment of the website. Several other studies have overcome the challenge of identifying usage information by utilizing a website that is password-secured for all the available content [44,52,54]. However, in this study, having a publicly available website prevented log-ins being created solely for participants. Thus, it is possible that the observations may have been attenuated because the intervention group consisted of participants who used the website minimally.

Another challenge a publicly available website as the intervention produced was the inability to structure the study as a randomized controlled trial. Participants were free to use the website, as were the public, at their own convenience. This challenge could have resulted in the observed improvements being driven by a self-administered “attention effect” due to the participants virtue for improvement, in which consumers may have had a strong desire to get better and were active in seeking ways to manage their disease as opposed to being solely driven by the intervention. Inversely, participants in the nonusers group could be unmotivated people who chose not to use the website and hence showed no improvements. Additionally, recruitment was via the Web and the online risk assessment using a self-reported screening tool potentially raises concerns of diagnostic misclassification. Furthermore, the use of the 2 self-reported outcome measures also resulted in a large number of analyses being carried out, which was not corrected for. This paper reports the raw outcomes of the instruments used (Multimedia Appendix 1).

A perceived barrier to the use of a Web-based resource to target older consumers is that they may not be well-versed with Internet usage. However, research has shown that 62% of respondents aged 55 years and older in Australia regularly use the Internet and have both the skills and equipment required to access health information online [55]. Although it is expected that Internet usage among older Australians will reach full saturation in several years, the redevelopment of the website still has to be conscious of the target audience in terms of language and design. However, despite full saturation being expected in several years, other existing means of self-management education, such as booklets, should be employed in conjunction with or parallel to the website to cater to those not using the Internet.

The study outcomes are positive and support the use of a Web-based platform for the dissemination of information and tools. The results of this study will help create a feedback summary for the refinement of the My Joint Pain website to help meet the needs of people at risk of OA or who have already developed this disease. We are now able to identify the gaps and strengths of the intervention that can influence redevelopment. Ideally, the education of patients through this website will improve joint decision making in the clinical consult and result in a higher treatment satisfaction for participants.

### Conclusion

An overburdened health care system, an aging population, and increasing Internet usage create the ideal construct for the development of Web-based health care resources. My Joint Pain users showed improvements in several aspects of quality of care received and the result can direct redevelopment to address limitations within the current layout and content of the website. Future studies should be carried out to evaluate its effectiveness on these domains and the website continuously reevaluated to meet the needs of the OA community.

## Appendix

Multimedia Appendix 1 Raw data from 0 and 12 month OA HUB evaluation.

## Abbreviations

heiQ: Health Evaluation Impact Questionnaire
HON: Health on Net
OA: osteoarthritis
OAQI: Osteoarthritis Quality Indicator
